# Finding Order in the Chaos: Outstanding Questions in Klebsiella pneumoniae Pathogenesis

**DOI:** 10.1128/IAI.00693-20

**Published:** 2021-03-17

**Authors:** Shekina Gonzalez-Ferrer, Hernán F. Peñaloza, James A. Budnick, William G. Bain, Hayley R. Nordstrom, Janet S. Lee, Daria Van Tyne

**Affiliations:** a Acute Lung Injury Center of Excellence, Division of Pulmonary, Allergy, and Critical Care Medicine, Department of Medicine, University of Pittsburgh School of Medicine, Pittsburgh, Pennsylvania, USA; b Division of Infectious Diseases, Department of Medicine, University of Pittsburgh School of Medicine, Pittsburgh, Pennsylvania, USA; c Department of Microbiology and Molecular Genetics, University of Pittsburgh School of Medicine, Pittsburgh, Pennsylvania, USA; d Veterans Affairs Pittsburgh Healthcare System, Pittsburgh, Pennsylvania, USA; University of California, Santa Cruz

**Keywords:** *Klebsiella*, evolution, innate immune system, pathogenesis

## Abstract

Klebsiella pneumoniae are Gram-negative facultative anaerobes that are found within host-associated commensal microbiomes, but they can also cause a wide range of infections that are often difficult to treat. These infections are caused by different pathotypes of K. pneumoniae, called either classical or hypervirulent strains. These two groups are genetically distinct, inhabit nonoverlapping geographies, and cause different types of harmful infections in humans. These distinct bacterial groups have also been found to interact differently with the host immune system. Initial innate immune defenses against K. pneumoniae infection include complement, macrophages, neutrophils, and monocytes; these defenses are primary strategies employed by the host to clear infections. K. pneumoniae pathogenesis depends upon the interactions between the microbe and each of these host defenses, and it is becoming increasingly apparent that bacterial genetic diversity impacts the outcomes of these interactions. Here, we highlight recent advances in our understanding of K. pneumoniae pathogenesis, with a focus on how bacterial evolution and diversity impact K. pneumoniae interactions with mammalian innate immune host defenses. We also discuss outstanding questions regarding how K. pneumoniae can frustrate normal immune responses, capitalize upon states of immunocompromise, and cause infections with high mortality.

## Abstract (precis)

Klebsiella pneumoniae are Gram-negative facultative anaerobes that are found within host-associated commensal microbiomes, but they can also cause a wide range of infections that are often difficult to treat. These infections are caused by different pathotypes of K. pneumoniae, called either classical or hypervirulent strains.

## INTRODUCTION

*Klebsiella* spp. are Gram-negative, nonmotile, rod-shaped bacteria that can live in a wide range of habitats (1). In humans, *Klebsiella* spp. can be found as commensals of the gastrointestinal tract, mouth, and nasopharynx (2). The *Klebsiella* genus contains over a dozen species, many of which cause opportunistic infections in humans (3). The K. pneumoniae species complex, including K. pneumoniae, K. quasipneumoniae, K. variicola, and K. africanensis, causes the highest burden of disease in humans (4, 5). Other species of *Klebsiella*, such as K. oxytoca and K. michiganensis, have historically been found less frequently during human infections (2); however, this appears to be changing (6, 7). Infections caused by K. pneumoniae include pneumonia, liver abscesses, bacteremia, soft tissue infections, urinary tract infections (UTIs), endophthalmitis, and meningitis (8). The reasons why *Klebsiella* spp. cause more frequent infections compared to other Gram-negative opportunistic pathogens are unclear. Possibilities include the bacteria’s ability to withstand starvation (9), naturally resist antibiotics (4, 10), outcompete other bacteria (11), readily exchange DNA with other members of the human microbiome (12), and acquire mobile genetic elements encoding a wide range of antibiotic resistance and virulence-enhancing genes (13).

Despite being one of the world’s most common nosocomial pathogens (14), K. pneumoniae display a population structure characterized by both abundant genetic diversity and the presence of a relatively small number of highly successful clonal genetic lineages (15). In contrast to other nosocomial pathogens, the most problematic K. pneumoniae clones (in terms of disease severity) are clearly divided into two phenotypically distinct groups that are characterized by either multidrug resistance or hypervirulence (16, 17). K. pneumoniae genomes usually contain 5,000 to 6,000 genes; however, the species pangenome, comprising both core genes (which are present in all strains) and accessory genes (which are variably present), is estimated to be greater than 100,000 different genes (4). Genetic lineages can be distinguished from one another based on their accessory gene content, and many of these accessory genes are present in only a small number of genomes. The abundant genetic diversity of K. pneumoniae clearly impacts the biology of the organism and, by extension, its interactions with mammalian hosts (18).

The most well-studied virulence factors in K. pneumoniae include the bacterial capsular polysaccharide (CPS), lipopolysaccharide (LPS), fimbriae, outer membrane proteins (OMPs), and iron-binding siderophores (8). Most K. pneumoniae strains produce a robust CPS that confers resistance to both antimicrobial peptides as well as phagocytosis by host immune cells (19, 20). Capsule composition and structure are highly variable between different K. pneumoniae lineages, and this variability translates into different levels of virulence. LPS, type 1 and type 3 fimbriae, and OMPs contribute to the bacteria’s ability to resist phagocytosis, affect adhesion to biotic and abiotic surfaces, and alter antibiotic permeability (18). Siderophores such as aerobactin, enterobactin, salmochelin, and yersiniabactin are secreted by K. pneumoniae, tightly bind to extracellular iron, and reenter the bacteria via specific import machinery (21). While siderophore content and expression are variable between different K. pneumoniae genetic lineages, it is clear that the ability to sequester iron contributes to the pathogenic potential of this organism (8).

The objective of this minireview is to discuss recent findings and unanswered questions regarding how K. pneumoniae interact with host innate immune defenses. To identify the manuscripts cited in this minireview, we performed keyword searches in the PubMed database and limited ourselves to discussing well-known and widely cited historic studies, or recent findings published within the last 5 years. A few terms will be utilized frequently throughout, and are explained in greater detail below. Hypervirulent K. pneumoniae (hvKp) cause fulminant disease, while classical K. pneumoniae (cKp) are less overtly pathogenic but can easily acquire multidrug resistance (MDR-Kp), including carbapenem resistance (CR-Kp). Regardless of these broad groupings, K. pneumoniae have evolved a vast array of different strategies that cause “chaotic” immune responses, allowing the bacteria to evade the mammalian innate immune system. Two principle axes of this immune evasion are resistance to complement-mediated killing and evasion of recognition and targeted clearance by host immune cells. Below we summarize what is currently known about how K. pneumoniae interact with the innate immune system, and how bacterial genetic diversity affects the pace and ultimate outcomes of these interactions.

## GLOBAL EPIDEMIOLOGY OF K. PNEUMONIAE INFECTION

**FIG 1:**
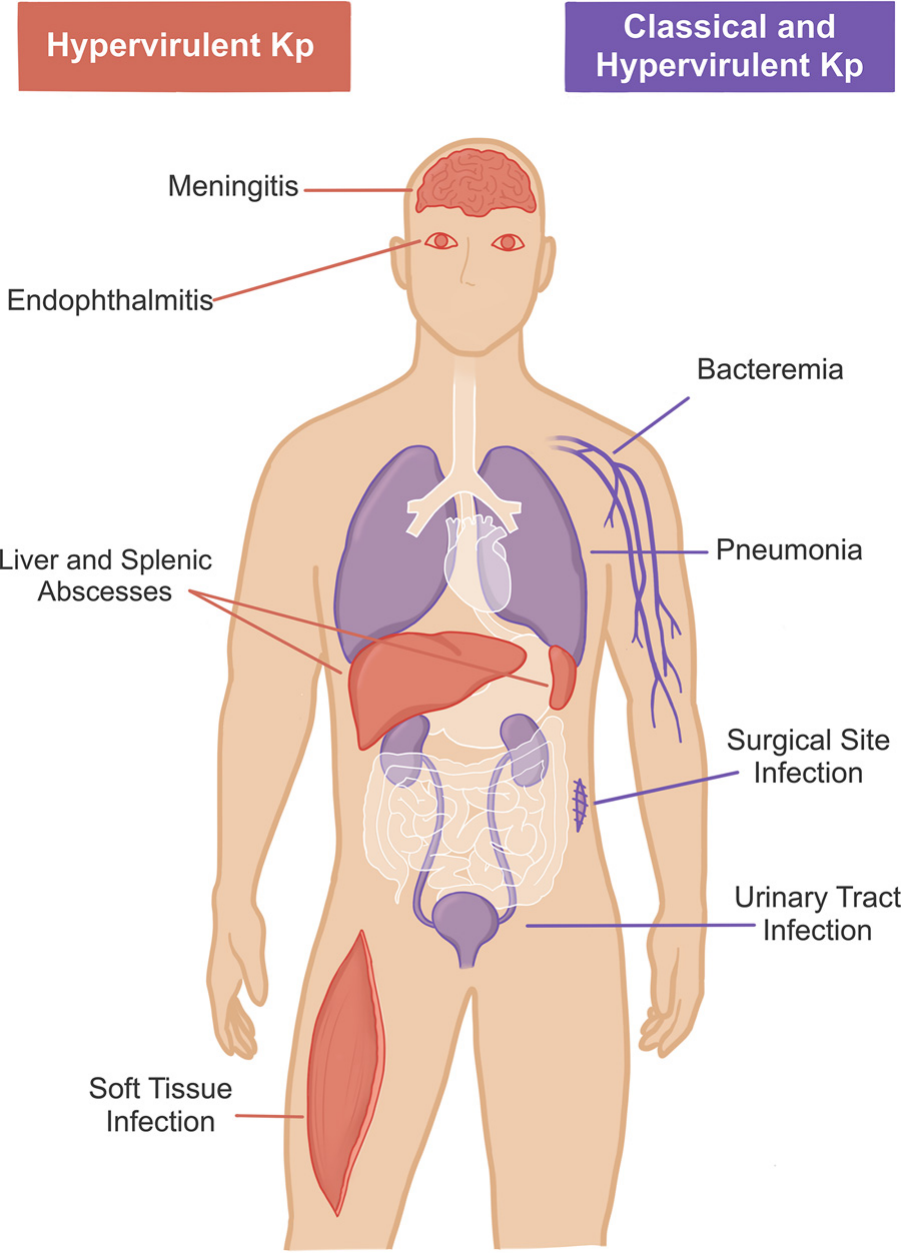
Anatomical sites of documented K. pneumoniae (Kp) infection. Hypervirulent K. pneumoniae infections (shown in red) are often community acquired and have been found to cause infections of the central nervous system, eyes, liver, spleen, and soft tissue. Infections with classical K. pneumoniae, which frequently evolve multidrug resistance, commonly arise in the hospital setting. Both classical and hypervirulent K. pneumoniae (shown in purple) have been found to cause bacteremia, pneumonia, surgical site infections, and urinary tract infections.

Since the discovery of K. pneumoniae (initially known as Friedlander’s bacillus) in the late 19th century, these bacteria have become global pathogens and have come to pose a significant threat to human health (17, 22). Within the last few decades, K. pneumoniae have been recognized as largely belonging to one of two distinct pathotypes—hvKp and cKp (17, 23). HvKp, first described in east Asia in the late 1980s, causes community-acquired invasive infections characterized by liver abscesses and metastatic infections of the lung, eye, central nervous system, musculoskeletal system, and urinary tract (24–27) (Fig. 1). While the term “hypervirulent” is frequently used by clinicians and researchers to describe K. pneumoniae, there is no molecular diagnostic nor microbiologic consensus for the definition of hypervirulence (28, 29). The classification of hvKp has emerged largely from clinical reports, leaving molecular diagnostic and microbiologic criteria for distinguishing between hvKp and cKp largely unestablished (17, 25, 30–33). There are nonetheless distinct clinical, epidemiological, and genetic differences between these two main pathotypes of K. pneumoniae.

In contrast to hvKp, cKp cause infections more frequently and commonly arise in the hospital setting; cKp frequently cause hospital-acquired pneumonia, UTIs, and bacteremia in immunocompromised patients with comorbidities (8, 30) (Fig. 1). CKp have extensively acquired mobile genetic elements that encode antimicrobial resistance genes (34). Extended-spectrum beta-lactamase (ESBL) and carbapenemase-encoding K. pneumoniae (together referred to as MDR-Kp) are globally disseminated and cause infections that are often difficult to treat, placing MDR-Kp high on current lists of significant threats to public health by the CDC and the WHO (17, 35, 36). The multidrug resistance of MDR-Kp and corresponding antimicrobial susceptibility of hvKp is an important distinction between these two pathotypes.

Two dominant typing schemes exist for classifying different types of K. pneumoniae. These include multilocus sequence typing (MLST), which is defined by the nucleotide sequences of seven housekeeping genes (*gapA*, *infB*, *mdh*, *pgi*, *phoE*, *rpoB*, and *tonB*), and determining the capsule type (K-typing) either by serotyping or sequencing of the *wzi* gene (37, 38). Both MLST and K-typing are common classifications for K. pneumoniae isolates for epidemiological purposes (8, 37). Sequence types (STs) commonly associated with MDR-Kp include ST11, ST258, and ST437, while STs commonly associated with hvKp include ST23, ST65, and ST86 (17, 23, 39). K-typing is frequently used to classify hvKp, where bacteria belonging to the K1 and K2 types are prevalent and cause invasive disease; however, not all K1 and K2 strains are hvKp (25, 26, 40). K1 and K2 serotype isolates often exhibit a hypermucoviscous phenotype, which can be defined semiquantitatively by the appearance of colonies exhibiting a viscous “string” of >5 mm in length on an agar plate (41). While sequence typing and serotyping are useful tools to track clonal isolates during outbreaks, they are not entirely accurate indicators of hypervirulence, as other serotypes aside from K1 and K2 have also been identified among hvKp isolates (30, 42, 43). Instead, it has been suggested that clinical diagnostics and epidemiologic surveys should focus on the accessory genomes of clinical isolates to more accurately identify and track hvKp (44).

Large-scale, whole-genome sequencing of K. pneumoniae in recent years has begun to reveal differences between hvKp and cKp on a genomic level. It is clear the accessory genomes of hvKp isolates are distinct from those of cKp, and that they contain genes related to increased virulence. These include genes that confer hypermucoviscosity, siderophores such as salmochelin and aerobactin, *peg344* (a putative transporter), the genotoxin colibactin, and operons encoding tellurite- and silver-resistance genes (17). While hypermucoviscosity is often attributed to increased capsule production, recent studies have shown that several genes involved in capsule formation have no effect on hypermucoviscosity, and vice versa, suggesting that hypermucoviscosity and capsule production are likely interrelated but distinct phenotypes (45, 46). The association of particular genotypes with the hypervirulence phenotype is clearly complex, as no single genetic marker alone can predict hypervirulence. Rather, hypervirulence phenotypes are likely the cumulative effect of different combinations of multiple accessory genes that work together to increase bacterial virulence.

From a diagnostic standpoint, however, the question remains: are there any commonalities within the genomes of hvKp isolates that can be used to accurately predict their hypervirulence phenotype? One study assessed the relationship between commonly associated genetic markers of hypervirulence and phenotypic analyses (including the string test and siderophore production) with epidemiological data and experimental virulence to determine a suite of biomarkers that could be used to identify hvKp isolates (30). The findings of this study showed that *peg-344*, *iroB* (salmochelin biosynthesis), *iucA* (aerobactin biosynthesis), and *_p_rmpA1* and *_p_rmpA2* (plasmid-borne regulators of mucoid phenotype), as well as the quantification of siderophore production, were accurately correlated with epidemiologic data and prediction of mortality in a murine model of sepsis compared to bacterial capsule type or the string test (30). The global application of these parameters has often led to the successful identification of hvKp (41, 47, 48). While there are clear limitations with this approach, further refinement of this genotype/phenotype approach could lead to more accurate and systematic identification of hvKp worldwide, as well as the identification of novel effectors of antibiotic resistance and hypervirulence phenotypes.

## CONVERGENCE OF hvKp AND MDR-Kp INTO HYPERVIRULENT BACTERIA THAT ARE HIGHLY RESISTANT TO ANTIBIOTICS

**FIG 2:**
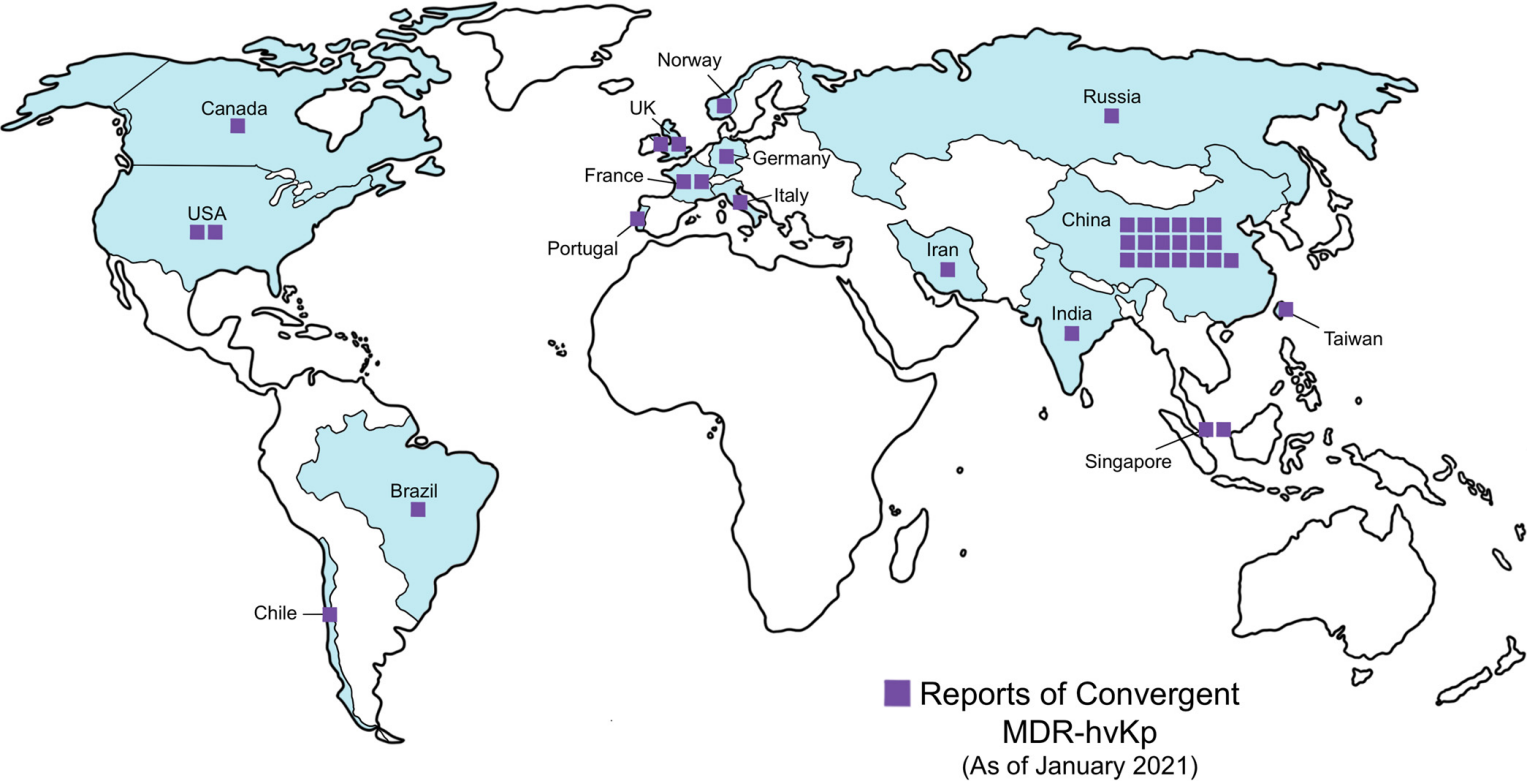
Global reports of infections with multidrug-resistant and hypervirulent K. pneumoniae (MDR-hvKp). Countries where convergent MDR-hvKp infections have been reported are shaded blue, and the number of reports from each country is indicated with purple squares. Reports represented are current as of January 2021.

The emergence and possible dissemination of K. pneumoniae isolates that possess both multidrug resistance and hypervirulence (MDR-hvKp) is a major concern. This concern is warranted, as there are already reported cases of MDR-hvKp from numerous countries, and these convergent events cause life-threatening infections that are difficult, if not impossible, to treat (49) (Fig. 2). Previous studies have thoroughly reviewed reports of convergent MDR-hvKp isolates from 2009 through early 2020, including genetic factors identified in the convergent isolates (50, 51). Since these studies were published, there have recently been additional reports of convergence in Asia, North America, Europe, and South America (52–61). Convergence occurs most frequently when mobile genetic elements carrying multiple antibiotic resistance genes (such as those encoding ESBLs and carbapenemases) are acquired by hvKp, or when virulence genes (like *rmpA* and siderophores), are acquired by MDR-Kp. The most alarming finding was the recent observation of mosaic plasmids that harbored both antimicrobial resistance and virulence genes in isolates obtained from patients in Norway, the United Kingdom, China, and Germany (56, 60–62), as these mosaic plasmids could act as a “one stop shop” for the simultaneous transmission of both hypervirulence and antimicrobial resistance phenotypes into cKp strains.

While the detrimental health impacts of convergent MDR-hvKp are high, the lack of a clear diagnostic approach may be one possible reason for the relatively small number of cases reported to date (50, 51). Another possibility is the presence of genetic barriers to the stable maintenance of convergent plasmids, including the fitness costs of gene acquisition (63). Furthermore, the acquisition of antibiotic-resistance genes and/or mutations can be detrimental to the virulence capabilities of pathogens in general (63, 64). This seems to be a plausible reason for the relatively sparse number of reports of infection caused by MDR-hvKp strains. It is conceivable that these isolates may be less fit than isolates that are solely MDR-Kp or hvKp, and thus they do not persist in the environment or colonize patients as readily. However, there is a general lack of knowledge about the fitness effects of transmission of resistance plasmids into hvKp isolates or virulence plasmids into MDR-Kp. By studying this process in greater depth, the actual threat of convergence can be better assessed.

Since convergent cases of MDR-hvKp include both hvKp that acquired resistance genes and MDR-Kp that acquired hypervirulence genes, this begs the question of whether a highly successful convergent strain or lineage would be more likely to occur in strains that are initially MDR-Kp or hvKp. One recent study compared the evolutionary dynamics of over 2,200 K. pneumoniae genomes from public databases (13). The authors found that MDR clones had greater pangenome diversity due to recombination events that led to the frequent acquisition and loss of mobile genetic elements compared with hypervirulent strains. In other words, MDR-Kp clones appear to more readily acquire virulence genes than do hvKp isolates. One possible reason for this might be that overproduction of CPS among many hvKp strains interferes with DNA uptake and inhibits the transfer of mobile genetic elements carrying resistance or virulence genes (13). This is supported by a study showing that a mutation in a capsule biosynthesis gene led to the evolution of hypervirulence in an MDR-Kp strain (65). These findings are troubling, as MDR-Kp strains are already endemic in many health care settings, and the widespread emergence of MDR-hvKp in these locations poses a significant public health threat. Altogether, convergent MDR-hvKp appear to be a looming threat to human health. While current attention focuses on characterizing the genetics and molecular basis of convergence, more robust surveillance will be key to accurately identify and monitor convergent strains on a global scale.

## COMPLEMENT EVASION AND SERUM RESISTANCE IN hvKp VERSUS MDR-Kp

**FIG 3:**
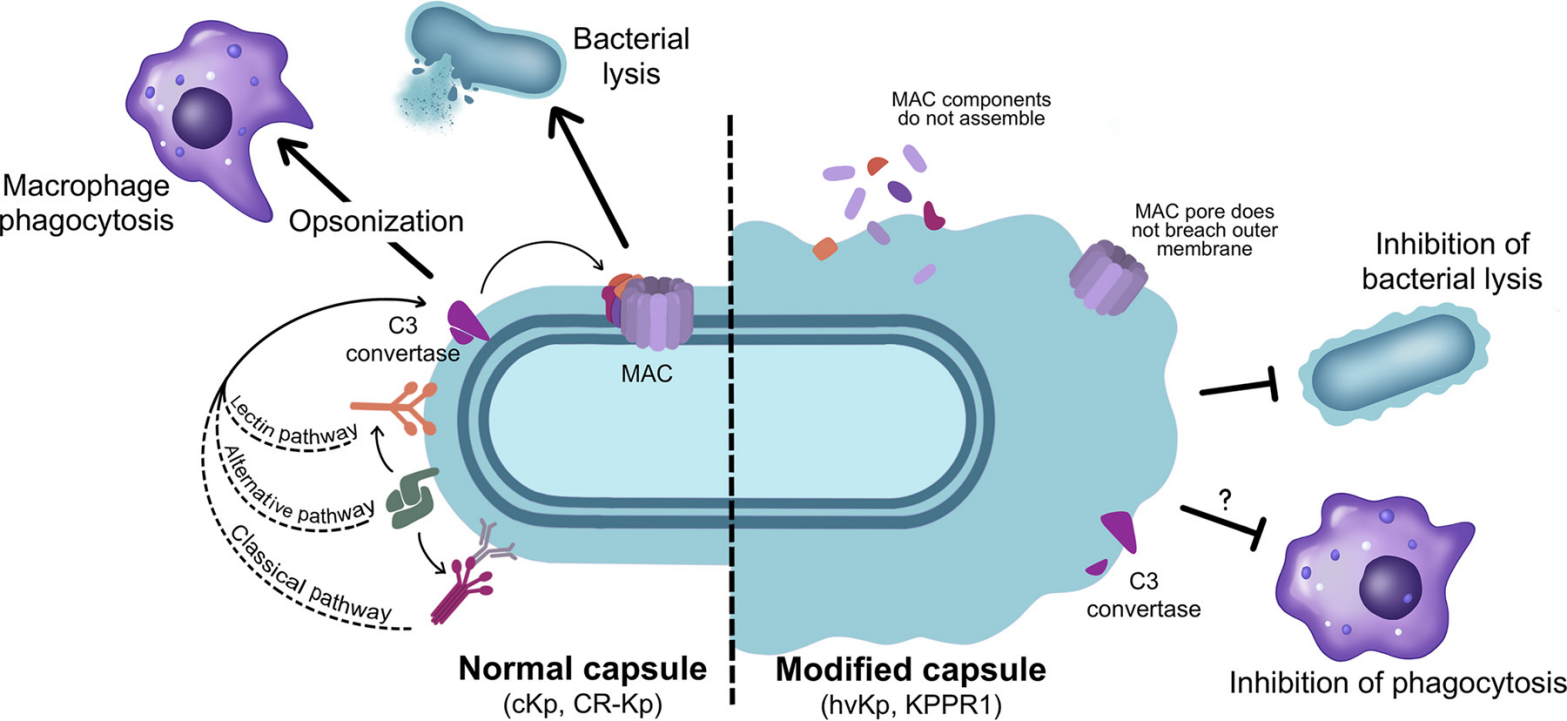
Complement evasion and serum resistance in K. pneumoniae. Differences between complement pathway activation and bacterial killing are depicted for K. pneumoniae with normal capsule (left) versus bacteria with modified capsule (right). Under normal conditions and with K. pneumoniae strains possessing a normal capsule (such as cKP and certain CR-Kp), the lectin, alternative, and classical complement pathways are able to recognize K. pneumoniae and recruit the C3 convertase, which leads to opsonization and phagocytic killing by macrophages and recruitment of the membrane attack complex (MAC). In contrast, some K. pneumoniae strains display a modified capsule (right); these include KPPR1, some hvKp isolates, and hypercapsulated CR-Kp isolates. The modified capsule in these strains is characterized by excess polysaccharide and changes in polysaccharide content and structure. These changes to the capsule cause resistance to complement-mediated killing through impaired recognition and binding of C3 convertase and MAC.

The complement system is an evolutionarily ancient component of the vertebrate innate immune system that is crucial for defending the host against pathogens, including K. pneumoniae. This system comprises more than 20 proteins present in vascular spaces and tissue microenvironments that coordinate with one another to quickly clear invading pathogens. The complement system supports host defenses against these invaders via two main mechanisms: (i) assembly of the membrane attack complex (MAC), consisting of C5b-C9 proteins, which can directly lyse the bacterial membrane, and (ii) opsonization of the bacterial cell surface to facilitate phagocytosis by immune cells (Fig. 3). There are three well-described pathways that can initiate the proteolytic cascade of the complement system: the classical, lectin, and alternative pathways. All three pathways converge on the formation of C3 convertases, which coordinate the opsonization and subsequent elimination of the pathogen. C3b in particular enables opsonization of bacteria by binding to amino or hydroxyl groups on the pathogen surface, and initiates MAC assembly via cleavage of C5 (Fig. 3).

The classical complement pathway is initiated when the pattern-recognition component of the C1 complex, called C1q, binds to antigen-antibody complexes. The lectin pathway is activated when host ficolin, collectin, or mannose-binding lectins (MBLs) recognize and bind to bacterial cell wall sugar motifs, such as d-mannose, which are readily found on K. pneumoniae. Lectin pathway recognition activates mannan-binding lectin serine proteases (MASPs) to initiate the complement cascade (66, 67). Conversely, the alternative pathway proteolytic cascade is constitutively active at very low levels, but pathway activation is amplified upon contact with “non-self” surfaces, such as bacterial membranes, through the C3 feedback loop (68). The alternative pathway is also notable for its role in amplifying the activity of both the classical and lectin pathways (68).

Both hvKp and MDR-Kp contain several factors that can activate different complement pathways, including LPS and O-antigen, OMPs, and CPS (69). LPS is able to activate the alternative pathway, and can also activate the classical pathway when lacking the O-antigen (70). OMPs such as OmpK36 interact with C1q of the classical pathway in an antibody-independent manner (69). CPS, which contains repeating units of d-mannose or l-rhamnose, is capable of activating the lectin pathway by direct interaction with MBLs (8, 69). Complement activation results in the formation of C3 convertase and subsequent cleavage of C3 to yield C3b, which can then opsonize the bacteria and initiate MAC assembly through formation of C5 convertases.

One of the primary mechanisms by which K. pneumoniae resist the host’s complement defense is through modification of the CPS. Several strains, including the classical lab strain KPPR1 (a derivative of the hypervirulent reference research strain of the K2 serotype, ATCC 43816) and other hvKp isolates, produce thick CPS that can inhibit MAC-induced lysis (69). One possible reason for this is that the CPS physically prevents the MAC C9 pore from breaching the bacterial membrane (69) (Fig. 3). Additionally, thick CPS may limit the recognition of other complement-activating surface molecules such as LPS, O-antigens, OMPs, and lectin-activating polysaccharide motifs (8, 69). Alternately, hvKp isolates can modify the composition of their CPS through variation of polysaccharide motifs, and can thereby bypass recognition by the lectin pathway (8, 69). The importance of the CPS to hvKp complement resistance and virulence is evident, and it continues to be a subject of frequent study in humans as well as *in vitro*.

Clinical studies have demonstrated the importance of CPS synthesis genes in the emergence of invasive K. pneumoniae infections that are prominent in Asia (25). Clinical isolates from these infections often display a hypermucoviscous phenotype, as well as resistance to normal human serum. Notably, hvKp isolates frequently carry the CPS biosynthetic gene mucoviscosity-associated gene A (*magA*; also known as *wzy*), as well as the regulator of mucoid phenotype A (*rmpA*) gene. Both of these genes have been shown to contribute to the bacteria’s ability to form thick capsules and cause invasive disease (25, 71). Genetic assays in laboratory settings have identified additional genes from both the core and accessory genomes that contribute to CPS-mediated complement resistance by hvKp (25, 67, 72, 73). For example, the transcription antiterminator *rfaH* is a core gene that was identified in a genome-wide screen as a prominent gene that contributes to K. pneumoniae fitness during lung infection (72). RfaH promotes the transcription of operons associated with virulence, such as CPS and LPS, and deletion of *rfaH* has been shown in multiple studies to impair the fitness of several hvKp strains (67, 72). Others have also shown that deletion of *hrtA*, a gene encoding a serine protease important for CPS formation, can lead to decreased CPS production, increased complement C3 binding, increased serum killing, and decreased virulence in mouse models of infection (69, 73). Numerous accessory genes that are not present in all K. pneumoniae strains, but that contribute to complement resistance in the strains that carry them, have also been identified (67). Overall, these studies suggest that hvKp have several mechanisms at their disposal to modulate CPS content and structure and thereby evade complement-mediated killing.

Beyond hvKp strains, there is growing concern about MDR-Kp, including CR-Kp (74, 75). Compared to hvKp, MDR-Kp isolates have been historically considered to be more sensitive to complement-mediated killing in human serum (76–78), and to be more readily bound by the MAC protein complex (67, 76). However, CPS synthesis is still thought to be essential for MDR-Kp fitness. For example, genetic variation at the CPS gene island has been fundamental in the evolution of the well-known carbapenem resistance-associated lineage called ST258. Within the ST258 lineage, two genetically distinct clades appear to have evolved through variation in the CPS locus (75). Furthermore, functional genomics studies have demonstrated that CR-Kp are susceptible to subtle genetic changes in CPS synthesis. For example, deletion of *rfaH* decreased capsule uronic acid production, increased complement C3b and MAC protein complex binding, and increased serum killing in a reference CR-Kp isolate called RH-201207 (67). The importance of the CPS in MDR-Kp fitness is further bolstered by a recent report that 64% of CR-Kp clinical isolates were able to persist in human serum, though they did not demonstrate the rapid growth that is typical of hvKp strains (78, 79). A more complete understanding of the role of CPS production and modulation in MDR-Kp is necessary to advance therapeutic strategies to limit the significant mortality associated with MDR-Kp infection in humans.

CR-Kp infections are associated with mortality rates ranging from 48 to 65% in several clinical series (80–82). Yet, MDR-Kp are relatively avirulent in immunocompetent mouse models of bloodstream infection, especially compared to hvKP strains (65, 75, 83). Among the potential reasons that could explain this discrepancy, there are two which may be beneficial to discuss for the purposes of focusing additional attention in the area. First, it seems plausible that CR-Kp may establish protected reservoirs in the human host that prevent eradication and allow for the accumulation of mutations that enhance virulence and, ultimately, increase morbidity and mortality. This hypothesis is supported by the enrichment of hypercapsulated isolates (encoding *wzc* mutations) found in bloodstream infections and low-capsule isolates (encoding *wbaP* mutations) found in the urinary tract (65). Interestingly, in this study, low-capsule strains demonstrated improved biofilm formation and increased invasion of bladder epithelial cells, suggesting possible fitness advantages for low-capsule mutants in the urinary tract (65). Furthermore, a small number of urinary CR-Kp isolates were found to be both hypercapsulated and more virulent in mouse models of infection. These hypercapsulated CR-Kp likely evolved from low-capsule strains that were initially present in the urinary tract, suggesting that persistence of low-capsule CR-Kp in “protected” tissue compartments can provide time for mutations to arise that confer a more virulent, hypercapsule phenotype.

A second possible reason for the discrepancy between CR-Kp virulence in different infection settings is variation in host complement defenses, which can affect the ability to clear bacteria from sites of infection. Differences in the host complement system likely increase susceptibility to K. pneumoniae infection in general, and more specifically to CR-Kp. This hypothesis is supported by the finding that both acquired and inherited complement deficiencies increase susceptibility to bacterial infections, especially those caused by encapsulated pathogens (84). Furthermore, mice that were deficient for C3 showed increased splenic dissemination following acute intrapulmonary infection with either the hypervirulent KPPR1 strain or a CR-Kp isolate (85). Deficiencies in the host complement system are particularly relevant in the intensive care unit setting, where K. pneumoniae infections are common and critically ill patients often exhibit acquired deficiencies in alternative complement pathway function (86). It was recently shown that decreased alternative pathway activity was associated with lower survival, more bloodstream infections, and impaired *in vitro* serum killing of CR-Kp in critically ill patients (85). Future research is clearly needed to elucidate the complex interplay between K. pneumoniae and the host complement system, including at the biophysical interface of the bacterial cell surface (87). In addition, wild-type cKp should be analyzed for their complement evasion strategies, as doing so might provide further clues to their widespread success. Such studies will likely lead to new strategies and therapies to counter the rising global health threat of K. pneumoniae infection (88).

## K. PNEUMONIAE INTERACTIONS WITH HOST INNATE IMMUNE CELLS

The complement system is not the only hostile immune element that K. pneumoniae need to overcome during an infection. Indeed, successful colonization of host organs by K. pneumoniae involves numerous interactions between the bacteria and different phagocytic cells, such as macrophages, neutrophils, and monocytes, which all share the primary goal of killing invading pathogens. K. pneumoniae possess several strategies to evade the antimicrobial activity of these cells, although the degree of success of immune evasion varies between KPPR1, hvKp, and CR-Kp strains. In this section, we focus on recent findings that highlight some of the major differences in how different K. pneumoniae strains interact with phagocytes from mice and humans *in vitro*, as well as *in vivo* in rodent models of infection.

**FIG 4:**
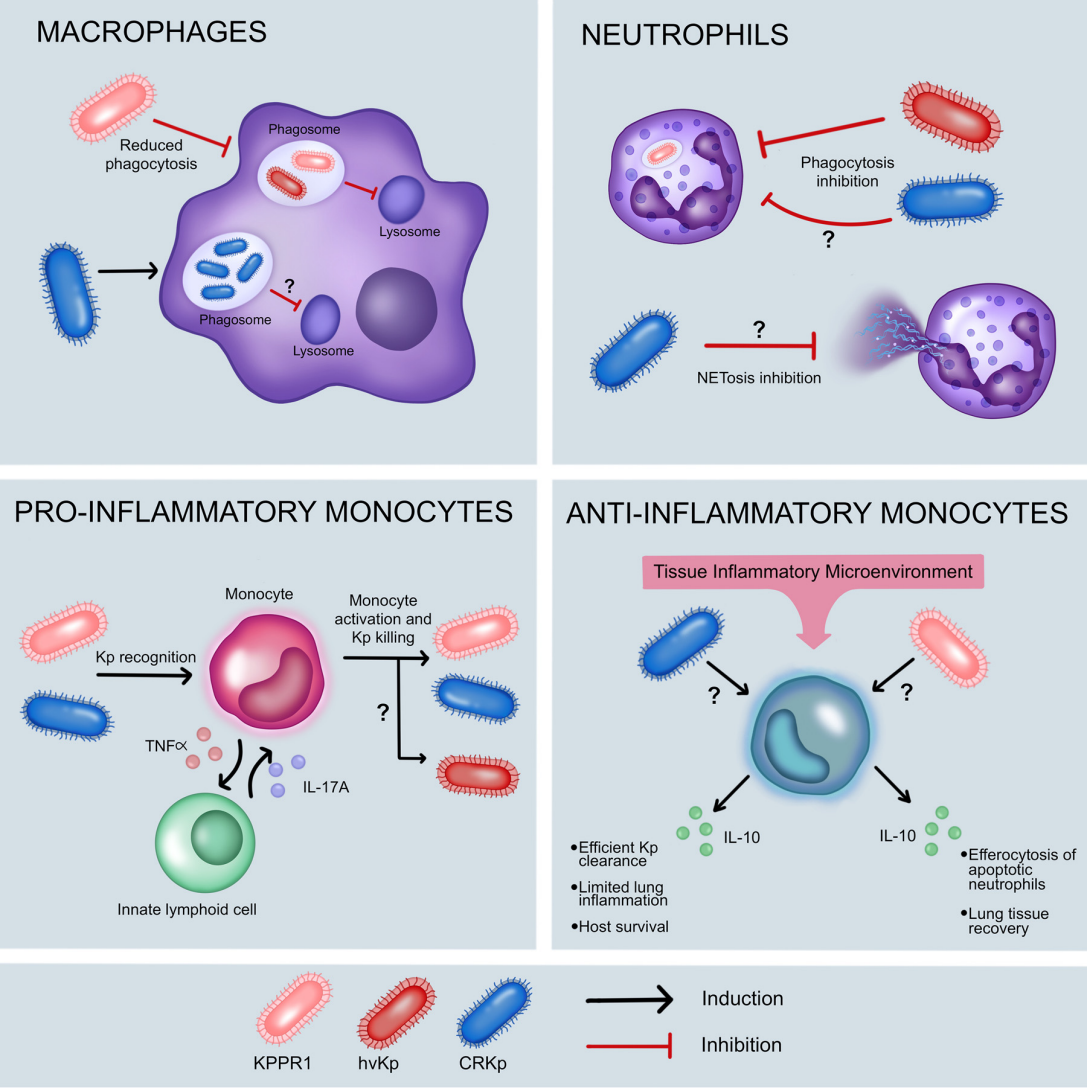
K. pneumoniae interactions with host innate immune cells. Each panel depicts our current understanding of how different K. pneumoniae strains interact with host innate immune cells, based largely on *in vitro* and animal infection studies. Differences between a widely used laboratory strain (KPPR1), hypervirulent clinical isolates (hvKp), and carbapenem-resistant isolates (CR-Kp) are shown. Black arrows show induction or activation, while red T-bars show inhibition. Question marks indicate interactions that remain to be fully elucidated. The depictions of proinflammatory and anti-inflammatory monocytes show how these cells have been observed to respond to K. pneumoniae in the lungs of mice during pneumonia. In the case of anti-inflammatory monocytes, K. pneumoniae infection promotes the expansion of these cells, which can either temper the inflammatory response and minimize lung injury at early time points during CR-Kp infection, or mediate the efferocytosis of apoptotic neutrophils and injury resolution at later time points during KPPR1 infection. In both cases, these anti-inflammatory effects are mediated by IL-10.

A prime example of the different pathogenic potential of different K. pneumoniae strains is the mortality associated with CR-Kp versus KPPR1 infection in mice. Whereas intraperitoneal and intratracheal infections with CR-Kp cause a sublethal infection, KPPR1 is rapidly lethal with equal inoculum (83, 89). Moreover, the 50% lethal dose (LD_50_) after intratracheal or intranasal infections ranges from 10^3^ to 10^4^ CFU for KPPR1, and from 10^6^ to 10^8^ CFU for CR-Kp (83, 89–91). The variation of LD_50_ values observed between KPPR1 and CR-Kp in rodent models of infection may, in part, be explained by differences in how these bacteria interact with and evade phagocytic cells (Fig. 4).

Macrophages are the initial phagocytes that encounter K. pneumoniae at mucosal sites, such as the lung (92, 93). They sense and activate mediators that drive appropriate immune responses, and typically eliminate K. pneumoniae following engulfment (Fig. 4). In this context, the evasion of the initial macrophage response, either by circumventing macrophage sensing and phagocytosis, or through resistance to intracellular killing, is essential for successful infection. *In vitro* studies have shown that murine and human macrophages can more efficiently phagocytose CR-Kp compared to KPPR1 (83, 94). Following engulfment, KPPR1 has also been reported to survive intracellularly within macrophages for hours, presumably by impairing phagosome maturation and phago-lysosome fusion through activation of the PI3K-Akt-Rab14 axis (94). How ingested KPPR1 manipulate the PI3K-Akt pathway and prevent phagosome maturation, and whether phagocytosed CR-Kp can survive within macrophages or are rapidly eliminated, remain unknown.

Alveolar macrophages play an important role in protecting the host against K. pneumoniae infection in the lung, as macrophage depletion during KPPR1-induced pneumonia in mice leads to increased mortality, increased lung bacterial burden, increased proinflammatory cytokine production, and excessive recruitment of neutrophils to the lung (95). Given that KPPR1 is poorly phagocytosed by macrophages, this finding suggests that the role of macrophages during KPPR1 infection is not restricted to phagocytosis and microbial killing, but also relates to their ability to exert regulatory functions to prevent an exaggerated immune response. However, it is also worth noting that macrophage depletion following CR-Kp pneumonia did not significantly affect host immune responses and mortality (90), suggesting that the initial signaling of alveolar macrophages required to drive a proper immune response can be compensated by other lung resident cells (such as lung epithelial cells), in the context of a less virulent K. pneumoniae infection (95).

Following infection in the lung, macrophages also function to limit extrapulmonary proliferation at tissue sites such as the liver and spleen. It was recently shown that severe KPPR1 infection heightens erythrophagocytosis by macrophages (a conserved innate immune response triggered by Toll-like receptor stimulation) and leads to suppression of interferon signaling through heme-mediated STAT1 dysregulation (96). As a consequence, macrophages were observed to acquire an immunosuppressive phenotype, which leads to an impaired ability of mice to control K. pneumoniae replication, exaggerated systemic inflammatory responses, and increased host mortality (96). This study highlights the importance of macrophages in controlling K. pneumoniae replication, and in regulating the inflammatory response at different tissue sites.

In addition to the role of macrophages, the rapid recruitment of neutrophils and monocytes is critical for effective control of K. pneumoniae through phagocytic killing (89, 90) (Fig. 4). Multiple reports have described the ability of hvKp isolates to evade neutrophil responses *in vitro* (97, 98). One of these studies also described a lower ability of human neutrophils to phagocytose serotype K1 and K2 hvKp isolates compared to cKp isolates with non-K1 or K2 serotypes and nonmucoid capsules (97). The other study confirmed these findings, and also showed that the evasion of neutrophil phagocytosis by K1 and K2 serotype hvKp isolates led to increased bacterial survival compared with nonmucoid cKp with non-K1 or K2 serotypes that lacked hypervirulence genes such as *rmpA* and aerobactin (98). This study also showed that hvKp failed to inhibit the release of neutrophil extracellular traps (NETs), which are networks of extracellular fibers composed of eukaryotic chromatin and other proteins extruded by dying neutrophils in response to pathogens (98). The study concluded that, among K1 and K2 hvKp isolates, the evasion of neutrophil-mediated killing is predominantly achieved by escaping phagocytosis, rather than the inhibition of the extracellular NET response. HvKp often produce excess CPS and display a hypermucoviscous phenotype (8), and it has been reported that a K1 isogenic noncapsular mutant was unable to escape neutrophil phagocytosis, suggesting that the evasion of neutrophil phagocytosis by hvKP is mediated, at least in part, by CPS (97).

While resistance to neutrophil phagocytosis has been observed among hvKp *in vitro*, the KPPR1 laboratory strain, as well as CR-Kp, show variability in their susceptibility to neutrophil-mediated killing. *In vitro*, murine neutrophils rapidly killed KPPR1 after 2 h of infection (90), and neutrophil depletion in a mouse pneumonia model impaired bacterial clearance and host survival, indicating that neutrophils are prominent in the immune response against KPPR1 (89). Data generated from *in vivo* experiments using a mouse pneumonia model have suggested that during CR-Kp infection, neutrophils are relegated to a secondary role in host defense. Indeed, the rapid recruitment of neutrophils during the first 48 h of infection did not necessarily correlate with a decrease in lung bacterial burden, which was detectable even at 7 to 10 days postinfection (90, 91). Although neutrophil depletion has been shown to have variable effects on lung CR-Kp burden, no significant effect on host survival was observed after neutrophil depletion (89, 90). *In vitro* studies using both murine and human neutrophils have allowed us to better understand, on a cellular level, the interaction between CR-Kp and neutrophils. For instance, one of the first *in vitro* studies that analyzed phagocytic killing by neutrophils showed that a major mechanism by which CR-Kp has adapted to evade neutrophil-mediated killing is through evasion of phagocytosis (99). A later *in vitro* study using a distinct CR-Kp isolate demonstrated that phagocytosed CR-Kp can disrupt phagosome acidification and survive intracellularly for at least 2 h in murine neutrophils (100). Finally, a recent study showed that a single CR-Kp isolate impaired granule mobilization and inhibited NET release by neutrophils (a process called NETosis) (101); however, the mechanism underlying this phenomenon is still to be described. Taken together, these studies suggest that CR-Kp possess several adaptations that allow them to evade neutrophil killing. These adaptations have likely helped relegate neutrophils to a secondary role in combatting CR-Kp *in vivo* in the lungs.

Other studies have found that the secondary role of neutrophils during CR-Kp infection is compensated for by monocytes, which are also rapidly recruited to the lungs during CR-Kp infection (89, 90). Monocytes are heterogeneous cells capable of displaying proinflammatory or immuno-regulatory phenotypes, depending on the nature of the microenvironment at the site of infection (102). Inflammatory CCR2^+^ monocytes are important cells in protecting the host during lung infection with both KPPR1 and CR-Kp, as their depletion was shown to lead to an impaired ability to clear K. pneumoniae from the lungs and resulted in elevated host mortality (89). During lung infection with CR-Kp, recruited inflammatory monocytes have been shown to induce interleukin 17 (IL-17) production by type 3 innate lymphocytes, which enhances the phagocytic and microbicidal activity of monocytes, thereby promoting efficient CR-Kp killing (103) (Fig. 4). On the other hand, anti-inflammatory monocytes, which are also referred to as monocytic myeloid-derived suppressor cells (M-MDSCs) (90, 91, 104), play a major role during K. pneumoniae lung infection, although their precise function seems to vary between CR-Kp versus KPPR1 infections (90, 91, 104) (Fig. 4). During CR-Kp infection in mice, M-MDSCs recruited to the lungs early during infection (i.e., within the first 24 h) promoted efficient bacterial clearance, protected the lung against tissue damage, and improved host survival through a mechanism involving IL-10 production (91). While excess IL-10 production early during infection may be detrimental during KPPR1 pneumonia in mice, cells that resembled M-MDSCs, or MDSC-like cells, were recruited to the lungs later during infection (i.e., 72 h), and appeared to play a beneficial role by mediating the clearance of apoptotic neutrophils by efferocytosis (104). The autocrine production of IL-10 by these MDSC-like cells increased neutrophil efferocytosis, which was critical to promoting lung recovery (104).

As MDSC-like cells are the main effectors that produce IL-10 during K. pneumoniae infection (91, 104, 105), KPPR1 appears to have also acquired the ability to subvert host responses by impairing the ability of MDSC-like cells to produce IL-10. During severe lung infection in mice, KPPR1 was observed to cause extensive immunopathology by impairing inflammation resolution through a mechanism involving SUMOylation (105). Specifically, KPPR1 pneumonia induced host cell death and the release of oxidized cardiolipin, a mitochondrial-derived phospholipid that is elevated in the lungs of humans and mice with severe bacterial pneumonia (105, 106). Extracellular oxidized cardiolipin induced accumulation of the metabolite cyclic phosphatidic acid (cPA, a potent antagonist of PPAR-γ), in MDSC-like cells and led to suppression of IL-10 production. This mechanism occurred through cPA-mediated SUMOylation of PPAR-γ, which prevented IL-10 transcription and tipped the cytokine balance toward persistent lung inflammation (105).

Overall, these studies highlight the heterogeneity in how KPPR1, hvKp, and CR-Kp interact with phagocytic cells and how they have evolved distinct ways to subvert host microbicidal responses. It is clear, however, that the outcomes of these interactions determine the tempo and ultimate result of host-pathogen interactions during K. pneumoniae infection. The bacterial factors involved in these interactions include CPS and LPS, among other structures largely present at the bacterial cell surface (107). Whereas *in vitro* studies analyzing the interactions between different hvKp isolates with neutrophils in particular show a common evasion strategy based on phagocytosis evasion, available data regarding CR-Kp show heterogeneous evasion strategies, including both phagocytosis evasion and prolonged intracellular survival. Large-scale studies using multiple and genetically diverse clinical isolates of cKp, MDR-Kp, and CR-Kp are needed to elucidate the common and unique strategies used by these different pathotypes to evade the innate immune system. Expanding our understanding of how K. pneumoniae genetic diversity impacts immune evasion would be the first step toward the development of new therapeutic strategies against these bacteria.

In conclusion, as highlighted in this review, there are many different factors that determine whether K. pneumoniae can successfully establish an infection and survive the selective pressures imposed by the host immune system. Two mechanisms that dominate this survival are the modification of bacterial cell surface structures and the acquisition of genes encoding new functionalities, such as hypervirulence and multidrug resistance. Whereas we might have once believed that these functionalities were carried by distinct genetic lineages, we now recognize they are highly plastic and can be readily gained or lost. While our current understanding of the genetic differences between K. pneumoniae isolates can explain some of the differences observed in innate immune tolerance and resistance, many questions remain unexplored.

Above all, there is a pressing need to focus on the dynamics and outcomes of bacterial-host interactions during K. pneumoniae infection in humans. Animal models and *in vitro* approaches have provided important evidence that the acquisition of hypervirulence and antimicrobial resistance by K. pneumoniae is linked to successful evasion of innate immune effectors. However, the successful adaptation of K. pneumoniae to these innate effectors, coupled with the fast-approaching “postantibiotic” era, necessitate a fuller understanding of how K. pneumoniae genotypes, antibiotic resistance, and hypervirulence mediate bacterial interactions with the human immune system. Some of the most important outstanding questions include the following. (i) Is convergence of MDR and hvKp indeed a true threat or a genetic anomaly? (ii) What are the collateral effects of complement resistance on bacterial growth and survival at sites of colonization, rather than sites of infection? (iii) How do cKp compare phenotypically and genotypically to the much more frequently studied hvKp and MDR-Kp subtypes? Further analysis of K. pneumoniae isolate genomes can potentially answer some of these questions, and will almost certainly uncover novel attributes yet to be described in this organism. Answering these questions will very likely facilitate the development of more accurate diagnostics tools and new therapeutic strategies targeted at the elimination of this dangerous pathogen.
